# Sensitive Detection of Plasma Fibrinogen Chain A mRNA in Hepatocellular Carcinoma Using Semi-Nested RT-PCR

**DOI:** 10.3390/diagnostics15111364

**Published:** 2025-05-28

**Authors:** Huy Duong, Minh Ngo, Trang Dao, Trang Hoang, Ung Nguyen, Tho Ho

**Affiliations:** 1 Department of Gastroenterology and Hepatology, 103 Military Hospital, Vietnam Military Medical University, Hanoi 100000, Vietnam; huyduonghvqy@gmail.com (H.D.);; 2 Radiology Center, 103 Military Hospital, Vietnam Military Medical University, Hanoi 100000, Vietnam; 3 Department of Genomics and Cytogenetics, Institute of Biomedicine and Pharmacy (IBP), Vietnam Military Medical University, No. 222, Phung Hung, Ha Dong, Hanoi 100000, Vietnam; 4 Department of Translational Clinical Research, Institute of Biomedicine and Pharmacy (IBP), Vietnam Military Medical University, Hanoi 100000, Vietnam; 5 Department of Microbiology, 103 Military Hospital, Vietnam Military Medical University, Hanoi 100000, Vietnam

**Keywords:** hepatocellular carcinoma, FGA mRNA, AFP (alpha-fetoprotein), semi-nested RT-PCR, cell-free mRNA

## Abstract

**Background/Objectives:** Hepatocellular carcinoma (HCC) remains a major cause of cancer-related mortality, with diagnostic limitations of existing biomarkers such as alpha-fetoprotein (AFP). This study evaluates plasma Fibrinogen chain A mRNA (FGA mRNA), alone and combined with AFP, for improving HCC diagnosis. **Methods**: A semi-nested RT-PCR assay was developed to quantify plasma FGA mRNA in 80 HCC patients and 74 controls (57 chronic liver disease [CLD] and 17 healthy donors [HDs]). Receiver operating characteristic (ROC) analysis was used to assess diagnostic performance, and logistic regression evaluated the combined biomarker model. **Results**: Plasma FGA mRNA levels were significantly higher in HCC patients than in CLD and HD controls (*p* < 0.0001). The area under the curve (AUC) for HCC vs. the combined control group (CLD + HD) was 0.721 (95% CI: 0.643–0.790), improving to 0.866 (95% CI: 0.782–0.927) when comparing HCC to HDs alone but declining for HCC vs. CLD (AUC = 0.678, 95% CI: 0.592–0.755). Combining FGA mRNA with AFP significantly enhanced diagnostic accuracy for HCC vs. CLD (AUC = 0.859, 95% CI: 0.790–0.913), with a sensitivity of 87.50% and specificity of 71.93%. In patients with low AFP levels (<20 ng/mL), the combined model identified 68.75% of HCC cases, outperforming AFP alone. **Conclusions**: FGA mRNA alone provides moderate diagnostic utility but substantially improves accuracy when combined with AFP, especially in low-AFP cases. This multi-biomarker approach holds promise for improving HCC detection and warrants further validation in larger cohorts.

## 1. Introduction

Hepatocellular carcinoma (HCC) is a global health burden, ranking as the sixth most common cancer and the third leading cause of cancer-related deaths worldwide, according to GLOBOCAN 2022 [1]. Despite advances in treatment, the prognosis remains poor due to late-stage diagnosis in most patients [2,3]. The early detection of HCC offers a critical window for curative interventions, yet current diagnostic modalities face significant limitations. Imaging techniques such as ultrasound, CT scans, and MRI, alongside serum biomarkers like AFP and PIVKA II, are commonly used but lack the sensitivity and specificity needed for detecting early-stage HCC. Liver biopsies, while more definitive, carry procedural risks, including bleeding and infection [4,5,6]. Hence, there is an urgent need for a more sensitive and non-invasive diagnostic tool.

Liquid biopsy, a method that analyses various circulating components in blood, including cell-free nucleic acids and circulating tumor cells (CTCs), has emerged as a promising alternative for cancer detection. By enabling the real-time monitoring of dynamic molecular changes, this approach addresses many limitations of conventional diagnostic techniques. Among these components, cell-free RNA (cfRNA) has gained attention for its ability to reflect tumor-specific genetic and epigenetic alterations, making it an attractive biomarker [7,8]. While cfRNA includes various RNA species, cell-free mRNA has recently emerged as a particularly valuable marker. Historically, mRNA was considered unsuitable for plasma-based diagnostics due to its susceptibility to rapid degradation by RNases [9]. However, recent advances reveal that mRNA in plasma is protected within membrane-bound structures such as exosomes, which shield it from degradation and facilitate its transport in the bloodstream [10]. This newfound stability has enabled the identification of specific mRNA markers, such as Fibrinogen Chain A (FGA) mRNA, which has been shown to significantly differentiate HCC from non-cancer controls in plasma cfRNA profiling studies [11].

Building on these findings, FGA mRNA levels were shown to be modestly higher in HCC patients compared to non-cancer controls, reflecting its potential as a biomarker for distinguishing between these groups. FGA mRNA not only exhibits liver-specific expression but is also closely tied to the coagulation and inflammation pathways—two processes critical to HCC progression. While the increase in FGA mRNA levels is statistically significant, it reflects a relatively small dynamic range of changes, necessitating highly sensitive methods for accurate detection. Detecting FGA mRNA in plasma is technically challenging due to its low abundance, subtle variations between patient groups, and the requirement for precise quantification in clinical applications.

To address these challenges, this study establishes a semi-nested reverse transcription polymerase chain reaction (RT-PCR) assay combined with DNA melting analysis for the accurate quantification of FGA mRNA in plasma. This method incorporates an internal control for normalization and quality assurance, enabling the precise detection of subtle variations in FGA mRNA levels. By validating this approach in HCC patients, this study investigates the clinical relevance of minor fluctuations in FGA mRNA, paving the way for its use as a non-invasive, highly sensitive tool for early cancer detection, disease monitoring, and personalized treatment strategies.

## 2. Materials and Methods

### 2.1. Subjects and Methods

This study recruited participants between February 2021 and May 2024 at the 103 Military Hospital, Vietnam Military Medical University. The cohort included 154 individuals, comprising 80 patients diagnosed with hepatocellular carcinoma (HCC) and 74 controls. Among the controls, 57 individuals had chronic liver disease (CLD), such as cirrhosis and chronic hepatitis, and 17 were healthy donors (HDs). The primary aim was to evaluate a novel quantitative RT-PCR assay for detecting circulating FGA mRNA.

HCC was diagnosed in accordance with the guidelines set by the American Association for the Study of Liver Diseases (AASLD 2023), with liver biopsies used to confirm any cases that were inconclusive. The stage of HCC was classified according to the Barcelona Clinic Liver Cancer (BCLC) staging system [4].

Participants with chronic hepatitis B were diagnosed according to the EASL 2017 Clinical Practice Guidelines on the management of hepatitis B virus infection [12]. The diagnostic criteria involved the detection of HBsAg in the serum for more than six months, alongside elevated serum HBV DNA levels, liver function tests indicating chronic liver inflammation, and hepatic fibrosis assessed by FibroScan. Liver cirrhosis was defined based on the Japanese Society of Gastroenterology (JSGE 2020) criteria, using clinical signs (jaundice, ascites, and hepatic encephalopathy), laboratory tests (serum albumin, bilirubin, and prothrombin time), and imaging techniques (ultrasound, transient elastography, and liver biopsy) [13]. Control participants were verified to be free of liver cancer through comprehensive physical examinations.

The CLD group was composed of patients with chronic hepatitis B or C infection and/or cirrhosis. Specifically, 47.4% (27/57) of CLD participants had cirrhosis, and 91.2% (52/57) had chronic hepatitis B virus (HBV) infection. The group also included a small number of patients with chronic HCV infection (5.3%) and/or alcohol-related liver disease. 

All participants were fully informed about this study’s objectives and provided consent, which ensured that this study’s findings were valid and reliable. This study was conducted according to the guidelines of the Declaration of Helsinki and approved by the institutional research committee of 103 Military Hospital, Vietnam Military Medical University (reference number: 12/2021/CNChT-HĐĐĐ).

### 2.2. Sample Collection and Processing

Approximately 10 mL of venous blood was collected from each participant into K2 EDTA tubes to prevent clotting, with plasma separated within six hours of collection and stored at −80 °C for subsequent analysis. To ensure sample integrity, blood specimens were visually inspected after centrifugation, and samples showing signs of hemolysis (e.g., pink or reddish discoloration) were excluded from this study. RNA was extracted from the plasma using the QIAamp Circulating Nucleic Acid Kit (Qiagen, Hilden, Germany), following the manufacturer’s instructions. The extracted RNA’s quantity and quality were assessed using a Nanodrop spectrophotometer to ensure suitability for downstream applications. Finally, the RNA samples were stored at −80 °C until further analysis.

AFP levels were measured at the Department of Biochemistry, 103 Military Hospital, using the Abbott ARCHITECT Ci16200SR immunochemistry analyzer (Abbott, Chicago, IL, USA). Serum alanine aminotransferase (ALT) and aspartate aminotransferase (AST) levels were assessed as part of routine liver function tests using the Olympus AU640 clinical chemistry analyzer (Olympus, Tokyo, Japan) at the same department.

### 2.3. Quantification of FGA mRNA

To accurately quantify circulating FGA mRNA in peripheral blood, a semi-nested RT-PCR assay was developed. This method employs two rounds of amplification to enhance sensitivity and specificity, with the sequences of primers and the synthetic internal control (IC) RNA detailed in Table 1.

#### 2.3.1. First Round of Amplification

**Figure 1:**
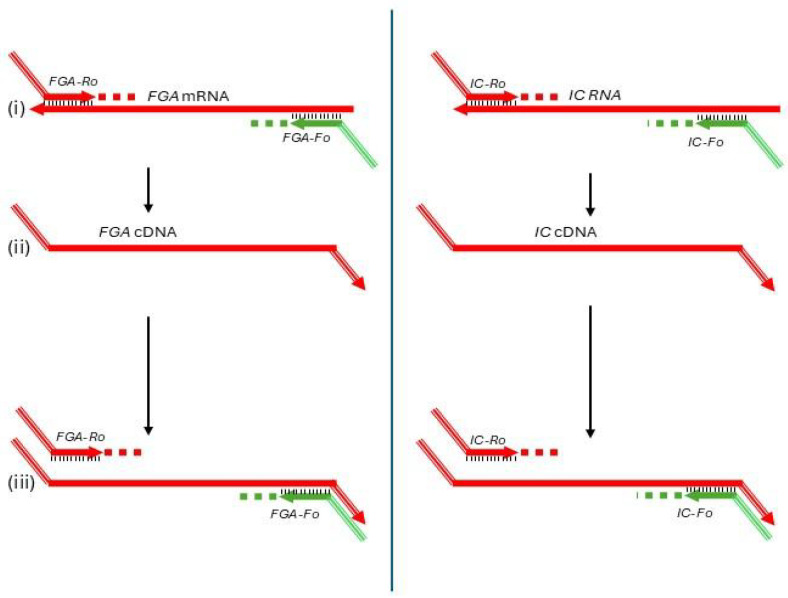
First round of the semi-nested RT-PCR assay for FGA mRNA quantification. This step amplifies FGA mRNA and a synthetic internal control (IC) RNA using specific primers (FGA-Fo, FGA-Ro, IC-Fo, and IC-Ro) with non-complementary 5′ ends to enable selective high-temperature annealing at 76 °C, ensuring specific cDNA amplification while preventing genomic DNA interference. Reverse primers also serve as templates for the common reverse primer (U-Ri) in the second round. Subfigures: (**i**) Primer annealing to mRNA; (**ii**) Reverse transcription extending the cDNA strand; (**iii**) PCR amplification of the resulting cDNA.

The initial round of amplification was designed to simultaneously amplify FGA mRNA and a synthetic RNA internal control (IC) gene (Figure 1). Specific primers for each target—FGA-Fo and FGA-Ro for FGA mRNA, and IC-Fo and IC-Ro for the IC RNA—were used (Figure 1i). To ensure specificity, the primers incorporated non-complementary sequences at their 5′ ends. These modifications allowed selective annealing at a high temperature (76 °C), which effectively amplifies cDNA synthesized from mRNA (Figure 1iii) while preventing amplification of contaminating genomic DNA, which can only bind to the primers at their short 3′ regions.

The reverse primers (FGA-Ro and IC-Ro) also included identical non-complementary sequences at their 5′ ends, creating a binding site for a common reverse primer (U-Ri) in the second round of amplification (Figure 1i). Additionally, these reverse primers acted as gene-specific primers for reverse transcription, ensuring the synthesis of cDNA from target RNA sequences (Figure 1ii).

The final master mix for the first round included primers at a concentration of 0.2 µM and 7.5 × 10^3^ copies of IC RNA per reaction. Template RNA extracted from plasma was added in a 2.5 µL volume per reaction. The amplification was carried out in a 1× HTOne Ultra RT-qPCR Probe master mix (HT Biotec, Ho Chi Minh city, Vietnam).

The amplification process was performed on an Applied Biosystems 9800 FAST Thermal Cycler (Thermo Fisher Scientific, Waltham, MA, USA). The thermal profile included the following: (1) Reverse transcription: 25 °C for 2 min, followed by 55 °C for 10 min; (2) Initial denaturation: 95 °C for 2 min; (3) Amplification cycles: One cycle at 95 °C for 15 s, 63 °C for 30 s, and 72 °C for 30 s, followed by 18 cycles at 94 °C for 15 s, 76 °C for 30 s, and 72 °C for 30 s; and (4) Final step: Hold at 4 °C indefinitely.

#### 2.3.2. Second Round of Amplification

**Figure 2:**
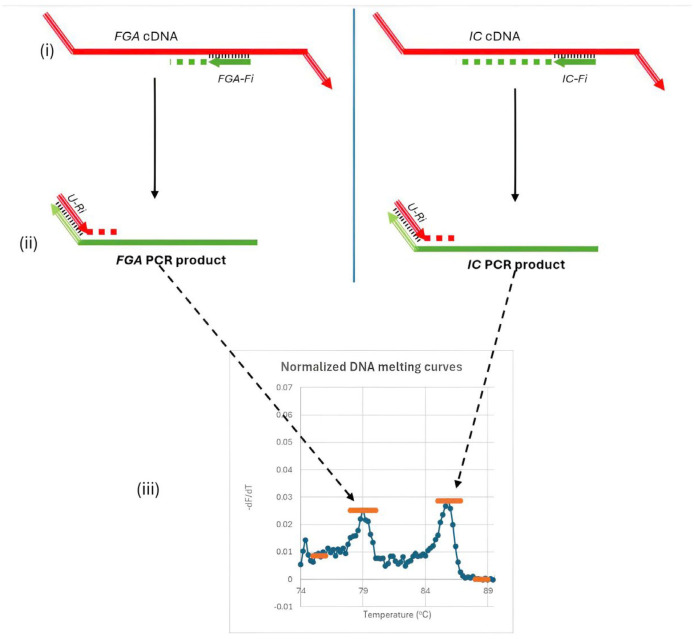
Second round of amplification and melting curve analysis in the semi-nested RT-PCR assay. This round uses specific forward primers (FGA-Fi, IC-Fi) and a common reverse primer (U-Ri) to amplify FGA mRNA and IC RNA efficiently. Distinct melting peaks for FGA (Tm ≈ 79.0 °C) and IC (Tm ≈ 86.1 °C) enable the quantification of FGA mRNA levels by calculating the ratio of FGA to IC peak heights. Subfigures: (**i**) Annealing of the nested forward primers (Fi); (**ii**) Binding of the common reverse primer (U-Ri); (**iii**) Signal detection and melting curve analysis.

The second round of the semi-nested RT-PCR assay refined the amplification process for FGA mRNA and the internal control (IC) RNA. Target-specific inner forward primers (FGA-Fi for FGA and IC-Fi for IC) and a shared reverse primer (U-Ri) were employed (Figure 2i,ii). The common reverse primer ensures consistent amplification efficiency for both target genes, enabling accurate quantification of FGA mRNA relative to the fixed IC concentration.

Each primer was used at a concentration of 0.2 µM, and the master mix is 1× HTOne MaX qPCR Green master mix (HT Biotec, Ho Chi Minh city, Vietnam). Amplification was performed on the Rotor-Gene Q instrument (Qiagen, Hilden, Germany) under the following conditions: an initial denaturation at 95 °C for 15 min, followed by 35 cycles at 94 °C for 15 s, 63 °C for 30 s, and 72 °C for 30 s. Post-amplification, a DNA melting curve analysis was conducted to identify distinct melting temperature peaks for the PCR products, with the target gene (FGA mRNA) yielding a Tm of approximately 79.0 °C and the IC exhibiting a Tm of approximately 86.1 °C (Figure 2iii). These distinct peaks allow precise differentiation between the amplified FGA and IC products.

The ratio of peak heights from the melting curve reflects the concentration of FGA mRNA relative to the IC. Small changes in FGA mRNA levels altered this ratio, facilitating the detection of subtle variations critical for early diagnosis and disease monitoring. The fixed IC concentration and the use of a common reverse primer ensure consistent amplification efficiency, enabling reliable detection of FGA mRNA even in low-abundance samples. The optimal amount of IC RNA (7.5 × 10^3^ copies per reaction) was determined experimentally through exploratory tests on plasma samples from HCC patients and non-cancer controls, ensuring the best differentiation between these groups.

Each RNA sample was analyzed in duplicate using the semi-nested RT-PCR assay. The concentration of FGA mRNA was calculated as the geometric mean of two technical replicates to ensure reproducibility and reduce variability.

### 2.4. Data Analysis

**Figure 3:**
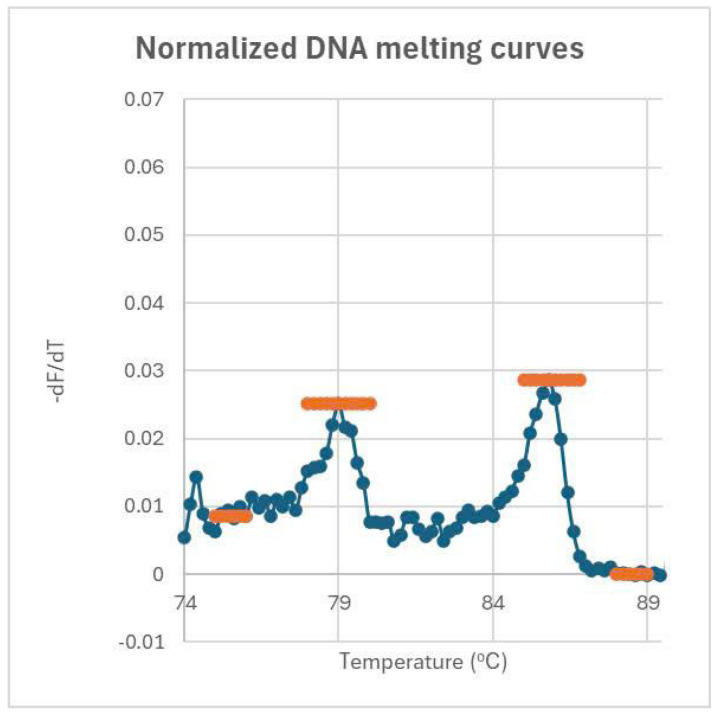
Normalized melting curves and peak height determination for FGA and IC genes. Peak heights are determined by subtracting the baseline height from the peak’s maximum height. The baseline for the FGA peak averages over 75–76 °C, while the baseline for the IC peak averages over 88–89 °C. The ratio of these peak heights reflects the concentration of FGA relative to IC.

The data obtained from the semi-nested RT-PCR assay were analyzed using a combination of quantitative and statistical methods to assess the diagnostic performance of plasma FGA mRNA. DNA melting curve analysis was used to calculate the peak heights of FGA mRNA and the internal control (IC) (Figure 3). The relative concentration of FGA mRNA was quantified by determining the ratio of the peak height of the FGA mRNA signal to the IC signal. The normalization of melting spectra and determination of baseline values ensured accurate quantification of FGA mRNA levels across all samples.

For diagnostic performance evaluation, ROC (receiver operating characteristic) curve analysis was conducted using MedCalc software to calculate the area under the curve (AUC), sensitivity, and specificity for plasma FGA mRNA, AFP, and their combination. The optimal diagnostic threshold for each marker was determined using the Youden index, which maximizes the sum of sensitivity and specificity. Additionally, logistic regression analysis was employed to develop and evaluate the combined biomarker model of FGA mRNA and AFP, facilitating comparisons with individual biomarkers.

For group comparisons, statistical significance was assessed using the Kruskal–Wallis test to evaluate overall differences in FGA mRNA levels among the three groups (HCC, CLD, and HD). When the overall test was significant, pairwise comparisons between groups were subsequently performed using the Mann–Whitney U test for non-parametric data.

Pairwise comparisons of ROC curves for individual and combined biomarkers were conducted to evaluate the added value of the combined model. Differences in AUCs were tested for statistical significance using Hanley and McNeil’s method.

All statistical analyses were performed using MedCalc software version 20.019 (MedCalc Software Ltd., Ostend, Belgium) with a significance level set at *p* < 0.05.

## 3. Results and Discussion

### 3.1. Study Population Characteristics

This study included 154 participants, comprising 80 hepatocellular carcinoma (HCC) patients and 74 controls, divided into 57 individuals with chronic liver disease (CLD) and 17 healthy donors (HDs). The demographic and clinical characteristics of these groups are summarized in Table 2.

The median age of the HCC group was significantly higher at 64 years (IQR: 54.3–69.8) compared to the CLD group (49 years, IQR: 41.0–61.5; *p* = 0.000) and HD group (43 years, IQR: 36.5–69.0; *p* = 0.004). Male predominance was observed across all groups, with 93.7% in the HCC group, 89.5% in the CLD group, and 76.5% in the HD group, but the gender distribution difference was not statistically significant (*p* > 0.05).

The HCC group exhibited significantly higher levels of ALT and AST compared to the HD group (*p* < 0.05), indicative of more severe liver dysfunction. The median AFP level in the HCC group (52.69 ng/mL, IQR: 5.57–944.05) was markedly higher than that in the CLD group (4.39 ng/mL, IQR: 2.80–6.96; *p* < 0.05), reflecting its diagnostic utility in distinguishing HCC from CLD.

Among HCC patients, 50% had cirrhosis, and 82.5% were positive for hepatitis B virus (HBV) infection. A smaller subset (12.5%) had alcohol-related liver disease. Most HCC cases presented with a single tumor (63.7%), with a median tumor diameter of 48.5 mm (IQR: 32.3–70.8). Advanced features such as vascular invasion (28.7%) and extrahepatic metastasis (5.0%) were observed in a subset of patients, underscoring the disease’s heterogeneity.

These detailed demographic and clinical profiles provide a solid foundation for evaluating the diagnostic performance of plasma FGA mRNA and its integration with AFP. The distribution of age, liver function parameters, and tumor-related features illustrates the biological variability within the cohort, reinforcing the need for advanced diagnostic tools to improve detection and management strategies for HCC.

### 3.2. Plasma Concentrations of FGA mRNA Across Patient Groups

**Figure 4:**
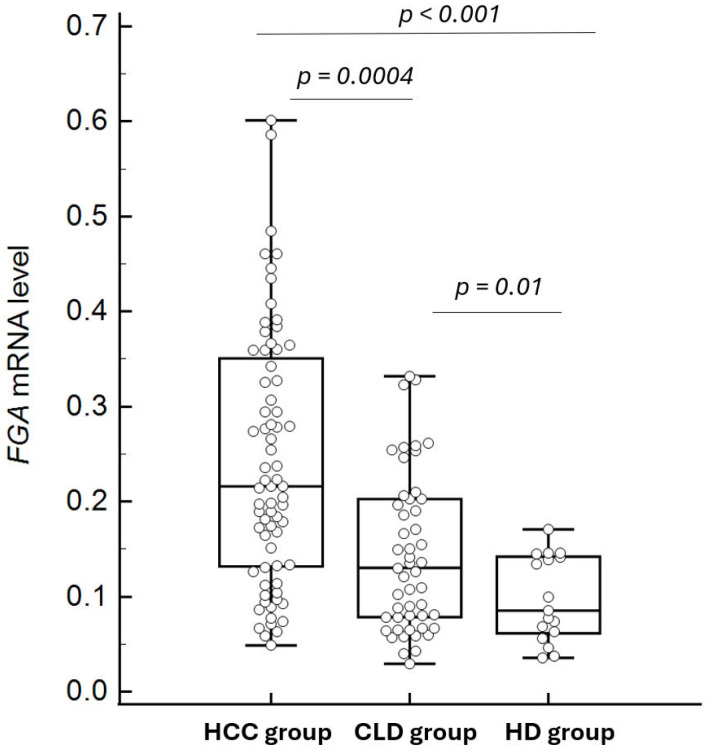
Comparison of FGA mRNA levels between HCC and control groups (chronic liver disease and healthy controls). The box plots depict the median and interquartile ranges of FGA mRNA levels in each group, showing significantly higher levels in the HCC group compared to both the chronic liver disease group (*p* = 0.0004) and the healthy control group (*p* < 0.0001). Some outliers in the HCC and CLD groups are not displayed to optimize the visualization of median and IQR comparisons.

Plasma FGA mRNA levels differed significantly among the three study groups, as determined by the Kruskal–Wallis test (*p* = 0.000001). Subsequent pairwise comparisons using the Mann–Whitney U test revealed that FGA mRNA levels were significantly higher in the HCC group compared to both the CLD group (*p* = 0.0004) and the HD group (*p* < 0.0001) (Figure 4). The median concentrations and their respective 95% confidence intervals (CIs) were as follows: HCC: 0.2366 (95% CI: 0.1972–0.2944), CLD: 0.1417 (95% CI: 0.1055–0.1925), and HD: 0.08527 (95% CI: 0.06314–0.1411). These results underscore the potential of FGA mRNA as a biomarker for distinguishing HCC patients from individuals with chronic liver diseases and healthy controls.

The findings demonstrate that plasma concentrations of cell-free FGA mRNA are significantly elevated in HCC patients compared to individuals with chronic liver diseases (CLD) and healthy donors (HDs). These results align with previous studies suggesting the potential of FGA mRNA as a biomarker for HCC [11]. The stepwise increase in FGA mRNA concentrations from HD to CLD and then to HCC underscores its association with liver pathology progression.

A key contribution of this study is the establishment of a semi-nested RT-PCR method for the quantification of FGA mRNA in plasma. This is the first report to develop and validate such a technique specifically for this biomarker. The semi-nested RT-PCR approach demonstrated the ability to reliably quantify FGA mRNA levels, providing sufficient sensitivity to differentiate HCC patients from both CLD and HD groups. The method’s capacity to detect subtle variations in FGA mRNA concentrations highlights its suitability for diagnostic purposes, addressing technical challenges often associated with low-abundance cell-free mRNA biomarkers.

The significant differences in FGA mRNA levels between HCC patients and controls highlight its diagnostic potential, despite some overlap with both CLD and HD groups. This study demonstrates the utility of semi-nested RT-PCR for quantifying FGA mRNA, supporting its integration into diagnostic workflows. The next section examines the diagnostic performance of plasma FGA mRNA, focusing on its sensitivity, specificity, and overall accuracy in differentiating HCC from control groups.

### 3.3. Diagnostic Performance of Plasma FGA mRNA

**Figure 5:**
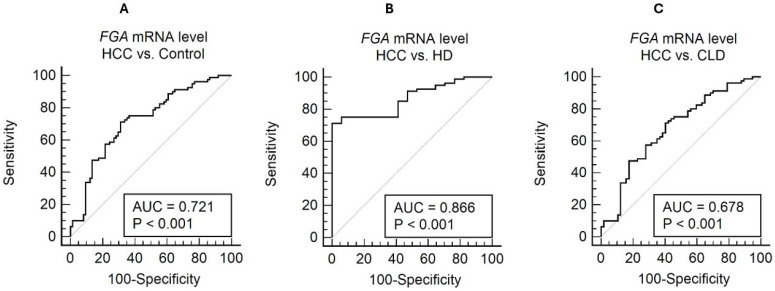
ROC curve analysis for cell-free FGA mRNA level in distinguishing HCC from non-HCC conditions. The ROC curves illustrate the diagnostic performance of FGA mRNA in three comparisons: (**A**) HCC vs. the combined control group (AUC = 0.721, sensitivity = 71.25%, specificity = 68.92%, and cutoff = 0.17); (**B**) HCC vs. healthy controls (AUC = 0.866, sensitivity = 71.25%, specificity = 100.00%, and cutoff = 0.17); and (**C**) HCC vs. chronic liver disease (AUC = 0.678, sensitivity = 71.25%, specificity = 59.65%, and cutoff = 0.17).

Plasma FGA mRNA demonstrated significant diagnostic potential in distinguishing hepatocellular carcinoma (HCC) from control groups (Figure 5), including chronic liver disease (CLD) and healthy donors (HDs). In comparisons between HCC and the combined control groups (CLD + HD), the area under the receiver operating characteristic (ROC) curve (AUC) was 0.721 (95% CI: 0.643–0.790) (Figure 5A). Using an optimal cut-off value of >0.17, determined by the Youden index, the sensitivity was 71.25%, and the specificity was 68.92%.

When comparing HCC with HD alone, the diagnostic performance improved significantly, with an AUC of 0.866 (95% CI: 0.782–0.927) (Figure 5B). The optimal threshold of >0.17 achieved a sensitivity of 71.25% and a specificity of 100%, highlighting FGA mRNA’s potential to clearly differentiate HCC from healthy individuals. However, in comparisons with CLD, the AUC was relatively lower at 0.678 (95% CI: 0.592–0.755) (Figure 5C). The sensitivity and specificity for this comparison were 71.25% and 59.65%, respectively, using a threshold of >0.17 (Youden index = 0.3090). These results underscore the challenge of distinguishing HCC from patients with chronic liver disease using FGA mRNA alone.

The diagnostic utility of FGA mRNA alone shows clear promise, but its limitations, particularly in differentiating HCC from CLD, highlight the need for integrated diagnostic strategies. This leads into the following section, which explores the combined use of FGA mRNA and AFP to enhance diagnostic accuracy.

### 3.4. Combined Use of Plasma FGA mRNA and AFP for Diagnosis

**Figure 6:**
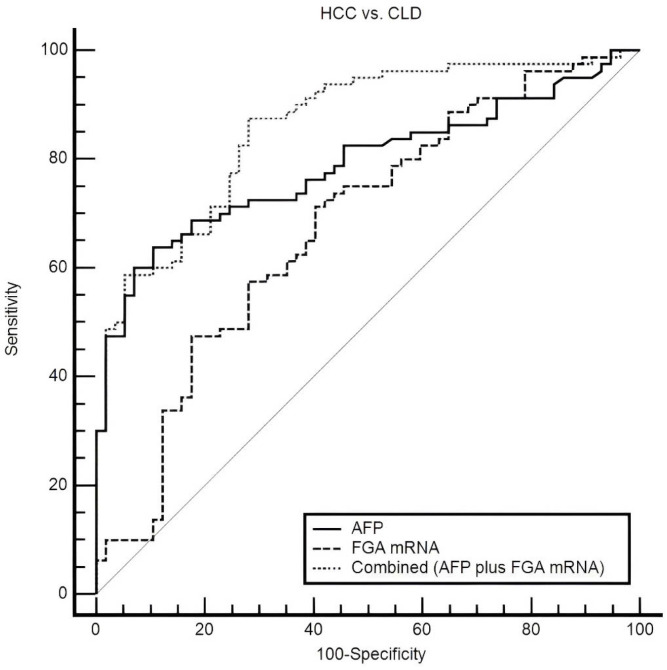
ROC curves comparing plasma FGA mRNA, AFP, and their combination in distinguishing HCC from CLD. The combined model achieves the highest AUC (0.859), outperforming AFP (0.789) and FGA mRNA (0.678). At the optimal threshold, sensitivity and specificity are 87.5% and 71.93%, respectively, highlighting the synergy of the combined approach.

The combination of plasma FGA mRNA and AFP demonstrated a marked improvement in diagnostic performance when distinguishing HCC from CLD, compared to the use of either biomarker individually (Figure 6). AFP alone showed a trend toward better diagnostic performance, with an AUC of 0.789 (95% CI: 0.711–0.854), compared to FGA mRNA. However, the difference between their ROC curves was not statistically significant (*p* = 0.0527). Integrating these biomarkers using a logistic regression-based model significantly improved the AUC to 0.859 (95% CI: 0.790–0.913), highlighting the complementary value of combining these markers and their potential for enhancing diagnostic accuracy. At the optimal threshold, the combined model demonstrated a sensitivity of 87.50% and specificity of 71.93%, offering a robust and balanced diagnostic profile. This balance is particularly critical in clinical practice, where minimizing both false negatives and false positives is essential for optimizing early detection and avoiding unnecessary interventions.

Statistical comparisons further supported the superiority of the combined model. It significantly outperformed AFP alone, with an increase in AUC of 0.0707 (95% CI: 0.0100 to 0.131, *p* = 0.0224), and demonstrated a substantial improvement over FGA mRNA, with an increase in the AUC of 0.182 (95% CI: 0.0949 to 0.269, *p* < 0.0001). These findings underscore the value of combining complementary biomarkers to address the limitations of individual markers and enhance diagnostic precision. AFP has long been recognized as a key biomarker for HCC, yet its sensitivity is often insufficient, particularly for early-stage disease or in patients with low AFP levels. Meanwhile, FGA mRNA provides additional diagnostic value by reflecting distinct molecular changes related to inflammation and coagulation pathways, processes critical to HCC progression.

The diagnostic performance of the combined marker was further evaluated across two AFP subgroups: HCC patients with elevated AFP levels (≥20 ng/mL) and those with low AFP levels (<20 ng/mL). In the high AFP group, the combined marker achieved perfect detection, correctly identifying 100% (48/48) of HCC cases, underscoring its robustness in cases where AFP alone already exhibits high sensitivity (Table 3). Notably, in the low AFP group, the combined marker significantly outperformed AFP alone, correctly identifying 68.75% (22/32) of HCC cases. This finding highlights the added value of the combined marker in addressing a critical diagnostic gap—HCC cases with low AFP levels, which are often missed when relying solely on AFP.

The limitations of AFP, particularly in detecting early-stage HCC or in low-AFP patients, are well documented [4,5,6]. While AFP remains a widely used biomarker, its sensitivity and specificity are insufficient for accurate standalone diagnosis [14]. In contrast, plasma FGA mRNA captures distinct molecular changes linked to systemic inflammation and coagulation pathways, processes critical to HCC progression. By combining these complementary biomarkers, the integrated model leverages distinct yet interrelated pathological mechanisms, offering significantly enhanced diagnostic precision.

This study builds on the foundational work of Roskams-Hieter et al. (2022), which identified plasma FGA mRNA as a potential biomarker for HCC using robust RNA sequencing and bioinformatic approaches [11]. Although their findings were groundbreaking, the small sample size of their study (8 HCC and 10 non-cancer controls) limited its generalizability. Our research advances this foundation by validating the diagnostic performance of FGA mRNA in a larger and more clinically diverse cohort using a semi-nested RT-PCR assay. This assay demonstrated acceptable diagnostic effectiveness, providing a cost-effective and accessible approach, particularly in resource-limited settings.

Fibrinogen, a glycoprotein synthesized in hepatocytes, consists of three protein subunits: FGA, FGB, and FGG. It plays a critical role in blood coagulation and systemic inflammatory responses, processes intricately linked to the progression of HCC [15]. The diagnostic relevance of FGA has been highlighted in several studies. Gustavo Ferrin et al. (2015) suggested that serum FGA protein levels, in combination with ceruloplasmin and paraoxonase 1, could serve as a diagnostic marker for HCC in hepatitis C virus-infected individuals [16]. Wei Dong et al. (2022) further demonstrated elevated levels of FGA protein in extracellular vesicles in the serum of HCC patients, emphasizing its potential diagnostic utility [17].

Gene expression analyses by Taiebe Kenarangi et al. (2022) and Xi Han et al. (2024) further identified FGA as part of the HCC gene signature, revealing decreased expression in tumor tissues compared to adjacent non-tumor tissues [18,19]. Lei Chen et al. (2024) also reported reduced expressions of FGA mRNA and protein in HCC tissues [20]. These consistent findings suggest that the elevated levels of FGA mRNA in the blood of HCC patients may originate from non-tumor liver tissues, where increased FGA synthesis could occur in response to systemic inflammatory and coagulation activity associated with HCC. This compensatory mechanism underscores the dynamic interaction between the tumor microenvironment and surrounding liver tissues, shedding light on the complex pathophysiology underlying elevated plasma FGA mRNA levels in HCC patients.

Despite its strengths, this study has several limitations that should be acknowledged. First, there was a notable age imbalance between the HCC and CLD groups, which may introduce potential confounding effects. Second, the number of healthy donors (HDs) included for comparison was relatively small, limiting the statistical power for subgroup analyses. Third, as a single-center pilot study, the generalizability of our findings remains restricted. These limitations underscore the need for further validation in larger, multi-center cohorts with balanced demographic characteristics and more extensive control populations. Nonetheless, the consistent trends observed across analyses provide a solid foundation for future investigations.

## 4. Conclusions

In conclusion, this study establishes a semi-nested RT-PCR assay to quantify plasma FGA mRNA, demonstrating its utility for hepatocellular carcinoma (HCC) diagnosis. Plasma FGA mRNA levels were significantly elevated in HCC compared to both chronic liver disease (CLD) and healthy donors (HDs), with the highest diagnostic performance observed for HCC vs. HD (AUC = 0.866). While FGA mRNA alone showed moderate accuracy (AUC = 0.678 for HCC vs. CLD), its combination with AFP significantly enhanced diagnostic performance, achieving an AUC of 0.859, sensitivity of 87.50%, and specificity of 71.93% for distinguishing HCC from CLD. Notably, the combined model was particularly effective in detecting HCC cases with low AFP levels, identifying 68.75% of cases missed by AFP alone. These findings highlight the potential of combining FGA mRNA and AFP as a robust diagnostic approach, particularly for addressing the diagnostic challenges of HCC vs. CLD. Future validation in larger cohorts is warranted to confirm its clinical applicability and refine multi-biomarker diagnostic strategies.

## Figures and Tables

**Table 1:** Primer, probe, and internal control (IC) sequences.

Amplification Round	Primers/Probe	Sequence (5′-3′)
1st PCR round	Forward primer (FGA-Fo)	CGACGTAAAACGACGGCCAGT-GAGAGGCGATTTTTCCTCAG
	Reverse primer (FGA-Ro)	CTGGCATATCATGACATACGACCTGA-CTGCTTCTCAGATCCTC
	Forward primer (IC-Fo)	CGACGTAAAACGACGGCCAGT-ACTAGCGTGCCTTTGTAA
	Reverse primer (IC-Ro)	CTGGCATATCATGACATACGACCTGA-GAGCGATACGAGCA
2nd PCR round	Forward primer (FGA-Fi)	ACCGTGATAATACCTAC
	Forward primer (IC-Fi)	CTCATTCGTTTCGGAAGAGC
	Reverse primer (U-Ri)	CTGGCATATCATGACATACGACCTGA
Internal control sequence (IC)		CGGCGTAATACGACTCACTATAGGGATGAACCGACGACGACTACAGCGTGCCTTTGTAAGCACAAGCTGATGAGTACGAACTTATGTACTCATTCGTTTCGGAAGAGCGGGCGGCTGCTCGCGGATACCCGTACCTCGGGTTTCCGTCTTGCTCGTATCGCTCGAGAACGCAAGTTCTGTTAACGTGAGTCTTGTAAAACCTTCTTTTTACGTTTACTCTCGTGTTAAAAATCTGAATTCTTCTAGAGTTCCTGATCTTC

Note: Underlined sequences are non-complementary and non-human-derived, ensuring amplification specificity.

**Table 2:** Demographic, laboratory tests, and tumor-related characteristics of study groups.

Characteristics	Category	HCC ^a^ (*n* = 80)	CLD ^b^ (*n* = 57)	HD ^c^ (*n* = 17)	*p*-Value
Gender	Male *n* (%)	75 (93.7%)	51 (89.5%)	13 (76.5%)	>0.05
	Female *n* (%)	5 (6.3%)	6 (10.5%)	4 (23.5%)	
Age (years)	<50	12 (15.0%)	29 (50.9%)	11 (64.7%)	<0.05
	50–59	16 (20.0%)	10 (17.5%)	1 (5.9%)	
	60–69	32 (40.0%)	10 (17.5%)	4 (23.5%)	
	≥70	20 (25.0%)	8 (14.1%)	1 (5.9%)	
	Median (IQR)	64.0 (54.3–69.8)	49.0 (41.0–61.5)	43.0 (36.5–69.0)	*p*(a,b) = 0.000 * p(a,c) = 0.004 *
PLT	Median (IQR)	181.5 (125.3–225.3)	153.0 (94.0–211.0)	-	-
Prothrombin %	Median (IQR)	86.0 (79.0–93.0)	-	-	-
INR	Median (IQR)	1.10 (1.05–1.17)	-	-	-
Albumin	Median (IQR)	37.7 (34.2–40.4)	-	-	-
Bilirubin total	Median (IQR)	13.7 (11.0–19.1)	15.0 (10.8–36.0)	-	-
AFP	Median (IQR)	52.69 (5.57–944.05)	4.39 (2.80–6.96)	-	*p* < 0.05
AST	Median (IQR)	55.2 (33.6–86.1)	54.1 (31.3–92.5)	27.6 (22.8–30.7)	*p* ^a,c^ < 0.05 *p* ^b,c^ < 0.05
ALT	Median (IQR)	43.6 (29.4–68.2)	45.5 (30.5–85.4)	25.6 (19.2–36.5)	*p* ^a,c^ < 0.05 *p* ^b,c^ < 0.05
Risk factor	Cirrhosis	40 (50.0%)	27 (47.4%)	0 (0%)	-
	HBV	67 (83.8%)	52 (91.2%)	0 (0%)	-
	HCV	1 (1.3%)	3 (5.3%)	0 (0%)	-
	Alcohol-related	10 (12.5%)	-	0 (0%)	-
Number of tumors	Single *n* (%)	51 (63.7%)			-
	Two *n* (%)	11 (13.8%)			-
	Three *n* (%)	4 (5.0%)			-
	More than three *n* (%)	14 (17.5%)			-
Greatest diameter (mm)	Median (IQR)	48.5 (32.3–70.8)			-
Tumor location	Right lobe *n* (%)	49 (61.2%)			-
	Left lobe *n* (%)	16 (20.0%)			-
	Both *n* (%)	15 (18.8%)			-
Vascular invasion	Yes	23 (28.7%)			-
	No	57 (71.3%)			-
Metastatic lymph node	Yes	3 (3.8%)			-
	No	77 (96.2%)			-
Extrahepatic spread	Yes	4 (5.0%)			-
	No	76 (95.0%)			-
BCLC stage	0	1 (1.25%)		-	-
	A	40 (50.0%)			
	B	12 (15.0%)		-	-
	C	26 (32.5%)		-	-
	D	1 (1.25%)		-	-

* Mann–Whitney Test. ^a^, ^b^, and ^c^ denote the HCC, CLD, and HD groups, respectively.

**Table 3:** Performance of the combined marker in detecting HCC cases across AFP subgroups.

AFP Level	HCC Detected by Combined Marker (Above Threshold)	HCC Missed by Combined Marker (Below Threshold)	Total
High AFP (≥20 ng/mL)	48	0	48
Low AFP (<20 ng/mL)	22	10	32
Total	70	10	80

## Data Availability

The datasets generated and/or analyzed during the current study, including individual patient data on circulating FGA mRNA levels for both hepatocellular carcinoma patients and control groups, are available from the corresponding author upon reasonable request. The aggregated and analyzed data are presented within the manuscript. Requests for access to the individual patient data should be directed to Dr. Tho Huu Ho at hohuutho@vmmu.edu.vn.

## Abbreviations

The following abbreviations are used in this manuscript:

HCC	Hepatocellular carcinoma
CLD	Chronic liver disease
HD	Healthy donor
AFP	Alpha-fetoprotein
FGA mRNA	Fibrinogen alpha chain messenger RNA
cfRNA	Cell-free RNA
RT-PCR	Reverse transcription polymerase chain reaction
ROC	Receiver operating characteristic
AUC	Area under the curve
IC	Internal control
BCLC	Barcelona Clinic Liver Cancer
ALT	Alanine aminotransferase
AST	Aspartate aminotransferase
HBV	Hepatitis B virus
HCV	Hepatitis C virus
IQR	Interquartile range
JSGE	Japanese Society of Gastroenterology
EASL	European Association for the Study of the Liver
